# Draft genome sequences of three multidrug-resistant *Klebsiella pneumoniae* strains isolated from alfalfa sprouts in Germany

**DOI:** 10.1128/mra.01270-25

**Published:** 2025-12-22

**Authors:** Tim Neumann, Annica Seemann, Denise Heß, Elisabeth Schuh, Michaela Projahn

**Affiliations:** 1 Department for Biological Safety, National Reference Laboratory for Escherichia coli including VTEC, German Federal Institute for Risk Assessment 27652, Berlin, Germany; University of Maryland Baltimore, Baltimore, Maryland, USA

**Keywords:** *Klebsiella*, sprouts, antibiotic resistance, ESBL, food

## Abstract

Multidrug-resistant *Klebsiella pneumoniae* are a threat to public health. Here, we present draft genomes of three recent *K. pneumoniae* isolates from alfalfa sprouts which reveal multiple resistance genes including extended-spectrum beta-lactamases and key virulence factors. Findings underscore the role of plant-based foods as potential reservoirs of clinically relevant bacteria.

## ANNOUNCEMENT

*Klebsiella pneumoniae* are opportunistic gram-negative bacteria of clinical significance, frequently associated with hospital-acquired infections (1, 2), but increasingly found in environmental and food sources (3–6). Their detection in ready-to-eat (RTE) foods raises concerns about potential transmission routes for multidrug-resistant strains to humans (7–10). As sprouts are often consumed without thermal processing, they can act as vehicles for pathogenic and antimicrobial-resistant bacteria (11–13).

In this study, we analyzed three *K. pneumoniae* isolates obtained from commercial alfalfa sprouts purchased from retail markets in Berlin, Germany, in April and June 2025. Sprout samples (25 g) were incubated in buffered peptone water at 120 rpm and 37°C. An aliquot of 100 µL of the overnight cultures was streaked on MacConkey Agar (Merck, Darmstadt, Germany) supplemented with cefotaxime (2 µg/mL) and incubated at 37°C for 24 h. Single colonies with typical morphology were isolated and identified by MALDI-TOF MS (Bruker Daltonics, Bremen, Germany). Genomic DNA was extracted from 4 mL overnight cultures (lysogeny broth) incubated at 37°C and 120 rpm using the PureLink Genomic DNA Mini Kit (Thermo Fisher Scientific, USA). Libraries were prepared with the Illumina DNA Prep (M) Tagmentation Kit (Illumina, San Diego, CA, USA) and sequenced on an Illumina MiSeq platform using the MiSeq Reagent Kit v3 (600-cycle) or the NextSeq benchtop sequencer using the NextSeq 500/550 Midoutput Kit v2.5 (300-cycle). Raw reads were trimmed and *de novo* assembled using the AQUAMIS pipeline v1.4.2 (https://gitlab.com/bfr_bioinformatics/AQUAMIS/), which implements fastp v0.23.2 for trimming (14) and shovill v1.1.0 (https://github. com/tseemann/shovill, based on Spades) for assembly. The AQUAMIS pipeline includes quast v5.2.0 for assembly quality control (https://github. com/ablab/quast).

Genome characterization was done with the BakCharak pipeline v3.1.6 (https://gitlab.com/bfr_bioinformatics/bakcharak) which includes multi-locus sequence typing (MLST) (https://github.com/tseemann/mlst), antimicrobial resistance (AMR) typing based on the NCBI AMRFinder (15), and virulence gene prediction using the virulence finder database(16).

The DNA isolation and sequencing kits were used according to the manufacturer’s instructions. Default parameters were used for all software tools.

Three different *K. pneumoniae* strains were isolated from alfalfa sprouts belonging to different MLST STs (Table 1). Strains of ST716, ST219, and ST189 were already isolated from human clinical specimens (17–20). All isolates carried multiple AMR genes (Table 1), including *bla*CTX-M-15, which is conferring extended-spectrum beta-lactam resistance and is associated with human infections worldwide (3, 21). Plasmid-mediated quinolone resistance determinants (*qnrB1/qnrS1*) were present in all strains (22). Furthermore, resistances to aminoglycosides (*aadA2*, *aph(3″)-Ib*, *aph (6)-Id*) and fosfomycin (*fosA*) were identified (23, 24). Virulence-associated determinants included *fimH* (type 1 fimbriae) and *mrkD* (type 3 fimbriae), supporting host colonization and biofilm formation (25). All isolates carried siderophore systems such as enterobactin (*entB*) and yersiniabactin (*ybt*). No high-virulence markers such as aerobactin locus (*iuc*) or genes responsible for hypermucoidy (*rmpA/rmpA2*) were detected, suggesting that these isolates do not represent hypervirulent lineages (6, 26) (Table 1).

**TABLE 1:** Data availability and key genomic features from the AQUAMIS assembly pipeline and the BakCharak analysis of the three *Klebsiella pneumoniae* isolates from sprouts

Feature	BfR-EC-20867	BfR-EC-20869	BfR-EC-20987
Sequence ID	25-EC00477-0	25-EC00479-0	25-EC00699-0
BioSample accession no.	SAMN51064586	SAMN51064588	SAMN51064596
SRA accession no.	SRR35254817	SRR35254816	SRR35254815
GenBank no.	JBROLS000000000	JBROLR000000000	JBROLQ000000000
No. of reads	1,370,298	1,938,111	2,271,682
No. of contigs	75	111	77
Q30 base fraction	0.933	0.941	0.877
Coverage depth	49.0	68.4	60.9
N50 (bp)	474,715	350,263	304,234
Total genome length (bp)	5,459,041	5,525,422	5,442,817
GC content (%)	57.06	57.15	57.35
MLST Warwick	ST716	ST219	ST189
Resistance genes			
Aminoglycosides	*aph(3″)-Ib*, *aph (6)-Id*	*aadA*2, *aph(3″)-Ib*, *aph(3′)-Ia*, *aph (6)-Id*	*aadA*2, *aph(3″)-Ib*, *aph (6)-Id*
Beta-lactams	*bla*CTX-M-15, *bla*SHV-27, *bla*TEM-1	*bla*CTX-M-15, *bla*SHV-1	*bla*CTX-M-15, *bla*SHV-80
Sulfonamides	*sul2*	*sul1*, *sul2*	*sul1*, *sul2*
Quinolones	*oqxA*, *oqxB19*, *qnrB1*	*oqxA10*, *oqxB5*, *qnrS1*	*oqxA*, *oqxB*, *qnrS1*
Trimethoprim	*dfrA14*	*dfrA12*	*dfrA12*
Fosfomycin	*fosA*	*fosA*	*fosA*
Tetracycline	/^*a*^	/^*a*^	*tet*(A)
*Klebsiella* key virulence genes (26–28)	*entB*, *fimH*, *iroE*, *iutA*, *KP1*, *mrkD*, *rfbK1*, *ybt*	*entB*, *fimH*, *iroE*, *iutA*, *KP1*, *mrkD*, *rfbK1*, *ybt*	*entB*, *fimH*, *iroE*, *iutA*, *KP1*, *mrkD*, *rfbK1*

^*a*^ /, no gene detected.

There is a particular risk of infection from consuming food contaminated with pathogens like *K. pneumoniae* (4, 6, 7). Especially, RTE foods or leafy greens and sprouts are of high concern. Our findings provide genomic evidence that sprouts for direct consumption harbor clinically relevant *K. pneumoniae* and therefore can be a risk for human health. This underlines the need for regular food safety monitoring and risk assessment.

## Data Availability

All data are encompassed under BioProject accession number PRJNA1315124. Sequencing raw reads were deposited in the NCBI Sequence Read Archive (SRA) (accession numbers: SRR35254815, SRR35254816, and SRR35254817), and draft genome files are available under GenBank accession numbers JBROLQ000000000, JBROLR000000000, and JBROLS000000000. The annotation of the draft genome files was added by the NCBI Prokaryotic Genome Annotation Pipeline (PGAP Version 6.10, 31 March 2025; https://www.ncbi.nlm.nih.gov/refseq/annotation_prok/) during upload.
